# Activity-Dependent Neuroprotective Protein (ADNP)-Derived Peptide (NAP) Counteracts UV-B Radiation-Induced ROS Formation in Corneal Epithelium

**DOI:** 10.3390/antiox11010128

**Published:** 2022-01-07

**Authors:** Grazia Maugeri, Agata Grazia D’Amico, Salvatore Giunta, Cesarina Giallongo, Daniele Tibullo, Claudio Bucolo, Salvatore Saccone, Concetta Federico, Davide Scollo, Antonio Longo, Teresio Avitabile, Giuseppe Musumeci, Velia D’Agata

**Affiliations:** 1 Section of Anatomy, Histology and Movement Sciences, Department of Biomedical and Biotechnological Sciences, University of Catania, 95100 Catania, Italy; graziamaugeri@unict.it (G.M.); sgiunta@unict.it (S.G.); g.musumeci@unict.it (G.M.); 2 Department of Drug Sciences, University of Catania, 95125 Catania, Italy; agata.damico@unict.it; 3 Department of Medical, Surgical Sciences and Advanced Technologies G.F. Ingrassia, University of Catania, 95123 Catania, Italy; cesarina.giallongo@unict.it; 4 Department of Biomedical and Biotechnological Sciences, Section of Biochemistry, University of Catania, 95123 Catania, Italy; d.tibullo@unict.it; 5 Pharmacology Section, Department of Biomedical and Biotechnological Sciences, University of Catania, 95123 Catania, Italy; claudio.bucolo@unict.it; 6 Center for Research in Ocular Pharmacology (CERFO), University of Catania, 95123 Catania, Italy; antlongo@unict.it (A.L.); t.avitabile@unict.it (T.A.); 7 Section of Animal Biology, Department of Biological, Geological and Environmental Sciences, University of Catania, 95123 Catania, Italy; saccosal@unict.it (S.S.); federico@unict.it (C.F.); 8 Department of Ophthalmology, Eye Clinic, University of Catania, 95123 Catania, Italy; davidescollo@hotmail.com

**Keywords:** corneal epithelium, UV-B, ADNP, NAP, ROS, apoptosis

## Abstract

The corneal epithelium, the outermost layer of the cornea, acts as a dynamic barrier preventing access to harmful agents into the intraocular space. It is subjected daily to different insults, and ultraviolet B (UV-B) irradiation represents one of the main causes of injury. In our previous study, we demonstrated the beneficial effects of pituitary adenylate cyclase-activating polypeptide (PACAP) against UV-B radiation damage in the human corneal endothelium. Some of its effects are mediated through the activation of the intracellular factor, known as the activity-dependent protein (ADNP). In the present paper, we have investigated the role of ADNP and the small peptide derived from ADNP, known as NAP, in the corneal epithelium. Here, we have demonstrated, for the first time, ADNP expression in human and rabbit corneal epithelium as well as its protective effect by treating the corneal epithelial cells exposed to UV-B radiations with NAP. Our results showed that NAP treatment prevents ROS formation by reducing UV-B-irradiation-induced apoptotic cell death and JNK signalling pathway activation. Further investigations are needed to deeply investigate the possible therapeutic use of NAP to counteract corneal UV-B damage.

## 1. Introduction

The cornea is a transparent and highly specialized tissue forming, along with the conjunctiva, the ocular surface. It protects the eye against different insults and provides two-thirds of the total refractive power of the eye [1]. The cornea comprises five different layers: The epithelium, Bowman’s membrane, the stroma, Descemet’s membrane, and the endothelium. The corneal epithelium acts as a dynamic barrier preventing the access of harmful agents to the intraocular space. It is a non-keratinized stratified squamous epithelium, constituted by 5–7 layers of cells forming a smooth uniform surface. The inner basal layer is characterized by a monolayer of columnar cells implicated in the generation of new suprabasal cells. Moreover, these cells secrete matrix factors essential for basement membrane and stromal function. The integrity of the cornea is dependent upon the self-renewing properties of limbal epithelial stem cells situated in crypts along the cornea-scleral border [2]. The cornea is subjected to different intrinsic and extrinsic insults, such as ultraviolet B (UV-B) irradiation, representing one of the most common corneal injuries. The cornea absorbs approximately 80% of UV-B rays, and prolonged exposure to this insult might provoke edema, photokeratitis, and photo-ophthalmia. The UV-B rays alter the corneal epithelium thickness and induce corneal endothelium damage causing fluctuating or occasionally blurred vision [3,4]. The UV-B irradiation drastically increases reactive oxygen species (ROS) levels, leading to apoptotic cell death [5,6]. More specifically, ROS produced by UV-B light act as second messengers to activate diverse redox-sensitive signalling transduction cascades, including the stress-activated MAP kinases p38 and the Jun-N Terminal kinase (JNK) [7]. The adverse effects of UV-B radiation can be reduced by the use of sunscreens, although total protection against this insult cannot be guaranteed. Damage to the corneal epithelium can result in pain, inflammation, vascularization, and in the worst-case, blindness. Based on the severity of the damage, keratoplasty may be needed. Therefore, the identification of molecules exerting a protective effect against UV-B-induced corneal damage might represent an effective alternative to the surgical approach. In our previous study, we demonstrated the beneficial effects of pituitary adenylate cyclase-activating polypeptide (PACAP) against UV-B radiation damage in human corneal endothelial cells [8]. PACAP is a well-established cytoprotective peptide, largely distributed in the central nervous system and in several peripheral organs [9,10]. It plays different functions through the activation of three distinct G-protein-coupled receptors: PAC1R, VPAC1R, and VPAC2R [11]. PACAP is involved in different biological processes such as cell division and survival [12,13,14] as well as exerting a protective role in neurodegenerative diseases [15,16,17,18,19,20].

Some PACAP effects are also mediated by the stimulation of an intracellular factor, known as the activity-dependent protein (ADNP) [21,22]. In 1999, Bassan et al. [21] synthesized a small peptide of eight amino acids derived from ADNP, known as NAP (davunetide, NAPVSIPQ/Asn-Ala-Pro-Val-Ser-Ile-Pro-Gln) acting as a protective agent in cerebral ischemia, severe head injury, and retinal damage induced by different insults [21,22,23,24,25,26,27]. It also exhibits metal chelating, antioxidant, and anti-inflammatory properties [28,29,30,31,32].

To date, the role of ADNP in corneal epithelium has not been investigated yet. Here we have demonstrated, for the first time, its expression in this tissue as well as the exogenous effect of ADNP mimicking peptide, NAP, on corneal epithelial cells exposed to UV-B radiations. Our results showed that NAP treatment reduces UV-B-irradiation-induced ROS formation, decreases JNK pathway activation, and consequently reduces apoptotic events. Further investigations are needed to determine the possible therapeutic use of NAP to counteract corneal UV-B damage.

## 2. Materials and Methods

### 2.1. Ethics Statement

This study was carried out according to the tenets of the Declaration of Helsinki. The human sclerocorneal button stored in organ culture at 31 °C were supplied for penetrating keratoplasty by the Eye Bank (Fondazione Banca degli Occhi del Veneto; Venezia-Mestre, Italy), which obtained informed consent for all tissue samples held and cultured (Ethical approval number 99/2019/PO).

### 2.2. Animals

All animal studies were in accordance with the Association for Research in Vision and Ophthalmology (ARVO) Statement for the Use of Animals in Ophthalmic and Vision Research. The animal protocols were reviewed and approved by the Institution of Animal Care and Use Committee of Catania University (Approval number 303). Male New Zealand albino rabbits (2.0–2.5 Kg; *n* = 7) were purchased from Envigo (Udine, Italy). The rabbits were housed under standard conditions. They were provided with food and water and maintained at a temperature and relative humidity of 21 ± 3 °C and 54 ± 4%, respectively. The lighting was artificial with a 12 h light/dark cycle, with lights switched on at 6 am. All animals were healthy and without ocular alterations.

### 2.3. Histological Analysis

Human and rabbit cornea were collected and fixed in 10% buffered formalin for 2 h. The specimens were washed overnight, dehydrated in graded ethanol, and paraffin embedded. The histological analysis was performed on 5 µm sections obtained using a rotary manual microtome (Leica RM2235, Milan, Italy). Cellular and tissue structure were viewed by staining the sections with H&E. The sections were examined using a Zeiss Axioplan light microscope (Carl Zeiss, Oberkochen, Germany) and the pictures were acquired with a digital camera (AxioCam MRc5, Carl Zeiss, Oberkochen, Germany).

### 2.4. Immunohistochemistry (IHC) Analysis

The expression and distribution of ADNP and p-63 in human and rabbit corneas was evaluated through immunohistochemical analysis [33]. The specimens were dewaxed in xylene, hydrated in graded ethanol and then incubated for 0.3% H_2_O_2_/methanol (30 min) to eliminate endogenous peroxidase activity. The sections were placed in a thermoregulated bath (80° for 30 min) with a rodent decloaker (Biocare Medical, Pacheco, CA, USA) to perform antigen retrieval. To reduce the non-specific binding of the antibody, the sections were blocked with 1% bovine serum albumin (BSA, Sigma, Milan, Italy) in PBS for 1 h, then the sections were incubated overnight at 4 °C with ADNP antibody (NBP1-89236; Novus Biologicals, Milan, Italy; 1:50) or rabbit p63 antibody (ab124762; Abcam, Cambridge, UK; 1:1000) work dilution in PBS and 1% BSA. The sections were incubated with secondary antibodies conjugated to polymer-HRP (LSAB+ System-HRP, K0690, Dako, Denmark), and the immunoreaction was detected by incubating them for 3 min in a 3,3′-diaminobenzidine solution (DAB substrate Kit; SK-4100, Vector Laboratories, Burlingame, CA, USA). The samples were lightly counterstained with hematoxylin, mounted in a vecta mount (Vector Laboratories) and observed with an Axioplan Zeiss light microscope (Carl Zeiss), and photographed with a digital camera (AxioCam MRc5, Carl Zeiss).

### 2.5. Cell Cultures

Statens Seruminstitut rabbit corneal (SIRC) epithelial cells (ATCC CCL-60) were grown in Eagle’s Minimum Essential Medium (ATCC^®^ 30-2003TM) supplemented with 10% fetal bovine serum (FBS, 10108-165, GIBCO, Milan, Italy) at 37 °C. The culture medium was exchanged every other day, and cultures were maintained until sub-confluence was reached. Cells were exposed to 30 s UV-B radiation at a dose of 20 mJ/cm^2^ using UVllink CL-508M (UVItec, Cambridge, UK).

### 2.6. Immunofluorescence Analysis

To determine the cellular distribution of the ADNP protein, immunofluorescence analysis was performed on SIRC cells as previously described by Maugeri et al. [34]. Cells cultured on glass coverslips were fixed in 4% paraformaldehyde in PBS for 15 min at room temperature, permeabilized with 0.2% Triton X-100, blocked with 0.1% BSA in PBS, and then probed with the rabbit anti-ADNP (NBP1-89236; Novus Biologicals; 1 µg/mL) antibody. Signals were revealed with Alexa Fluor 488 goat anti-rabbit, for 1.5 h at room temperature (shielded from light). DNA was counterstained with 4,6-diamidino-2-phenylindole (DAPI; cat. no 940110; Vector Laboratories, Burlingame, CA, USA). After a series of washes in PBS and double-distilled water, the fixed cells were cover-slipped with the Vectashield mounting medium (Vector Laboratories). Immunolocalization was analyzed by confocal laser scanning microscopy (Zeiss LSM700, Oberkochen, Germany) at 40×.

### 2.7. Cell Viability Assay

Cell viability was determined by 3-[4,5-dimethylthiazol-2-y l]-2,5-diphenyl tetrasodium bromide (MTT assay, cell proliferation kit, catalog number: 11465007001, Roche Diagnostics, Indianapolis, IN, USA). Cells were seeded into 96-well plates at a density of 1 × 10^4^ cells/well in 100 μL of the culture medium for 1 day [35]. After overnight growth, SIRC cells were grown in the different experimental conditions, and at the end of treatment, 0.5 mg/mL of MTT was added to each well and incubated for 4 h at 37 °C. The reaction was stopped by adding 100 μL of the solubilization solution, then formazan formed by the cleavage of the yellow tetrazolium salt MTT was measured spectrophotometrically by an absorbance change at 570 nm in a plate reader (VariosKan, Thermo Fisher Scientific, Waltham, MA, USA). Twelve replicate wells were used for each group. The medium alone was used as a blank.

### 2.8. Fluorescence Microscopic Analysis of Cell Death

Cells were seeded into 12-well plates at a density of 4 × 10^3^ cells/well in 500 μL of the culture medium. After 24 h, cells were incubated in the control medium (CTRL), or in the presence of 10 nM NAP, or exposed to ultraviolet irradiation (UV-B) for 30 s with or without NAP for 24 h. Cells were incubated with a solution of methanol/acetic acid (3:1 *v/v*) for 30 min, rinsed three times in PBS, and incubated with Hoechst 33,342 dye (0.4 μg/mL). When all the necessary washing steps were completed, cells were visualized using the Axiovert 40 fluorescence microscope (Carl Zeiss, Oberkochen, Germany) as previously described by Maugeri et al. [36]. Apoptotic cells were recognized on the basis of nuclear condensation and/or fragmented chromatin. Each condition was reproduced in three dishes per experiment. Both apoptotic and normal cells were counted from three fields per dish in a fixed pattern. The apoptotic nuclei counting was carried out independently by two investigators, and wherever disagreement arose, a third investigator was called upon to verify the results.

### 2.9. Detection of ROS

ROS was measured by means of the 2′,7′ –dichlorofluorescin diacetate (DCFDA)–Cellular Reactive Oxygen Species Detection Assay Kit (ab113851, Abcam, Cambridge, UK), according to the manufacturer’s protocol. SIRC cells were plated into 96-well black plates at a density of 1 × 10^4^ cells/well for 24 h. After overnight growth, SIRC cells were grown in the control medium (CTRL), or in the presence of 10 nM NAP, or exposed to ultraviolet irradiation (UV-B) for 30 s with or without NAP for 24 h. Cells were washed gently in PBS twice and incubated with 25 μM DCFDA previously dissolved in a buffer solution for 45 min in the dark. The determination of the ROS concentration was performed by measuring the DCF fluorescence (λex = 495 nm, λem = 529 nm) with VarioskanTM. Twelve replicate wells were used for each group.

Mitochondrial-derived ROS (mtROS) detection was performed using the MitoSOX™ Red mitochondrial superoxide indicator (ThermoFisher Scientific, Milano, Italy). Cells were stained using 3 µM of the MitoSOX™ Red mitochondrial superoxide indicator (ThermoFisher Scientific, Milano, Italy). After incubation for 10 min at 37 °C, cells were washed three times and analyzed by MACSQuant Analyzer 10 (Miltenyi Biotec, Bologna, Italy).

### 2.10. Western Blot Analysis

Western blot analysis was performed according to the procedures previously described [37]. Proteins were extracted with buffer containing 20 mM Tris (pH 7.4), 2 mM EDTA, 0.5 mM egtazic acid, 50 mM mercaptoethanol, 0.32 mM sucrose, and a protease inhibitor cocktail (Roche Diagnostics, Monza, Italy) using a Teflon-glass homogenizer and then sonicated twice for 20 s using an ultrasonic probe, followed by centrifugation at 10,000× *g* for 10 min at 4 °C. Protein concentrations were determined by the Quant-iT Protein Assay Kit (Invitrogen, Carlsbad, CA, USA). About 20 μg of protein homogenate were diluted in 2 × Laemmli buffer (Invitrogen), heated at 70 °C for 10 min, and then separated on a Biorad Criterion XT (Hercules, CA, USA) 4% to 15% bis-tris gel (Invitrogen) by electrophoresis and then transferred to a nitrocellulose membrane (Invitrogen). Blots were blocked using the Odyssey Blocking Buffer (Li-Cor Biosciences, Nebraska, NE, USA). The transfer was monitored by a prestained protein molecular weight marker (BioRad Laboratories, Milan, Italy). Immunoblot analysis was performed by using appropriate antibodies: Rabbit anti-ADNP (NBP1-89236; Novus Biologicals; 1:50); mouse anti-Bcl2 (sc-509, Santa Cruz Biotechnology, Dallas, TX, USA; 1:200); mouse anti-Bax (sc-20067, Santa Cruz Biotechnology; 1:200); rabbit anti-JNK (sc-571; Santa Cruz Biotechnology, 1:200); mouse anti-p-JNK (sc-6254; Santa Cruz Biotechnology, Texas City, TX, USA; 1:200); and rabbit anti-β-tubulin (cat n.sc-9104, Santa Cruz Biotechnology; 1:500). The secondary antibodies goat anti-rabbit IRDye 800CW (926-32211; Li-Cor Biosciences) and goat anti-mouse IRDye 680CW (926-68020D; Li-Cor Biosciences, Nebraska, USA) were used at 1:15,000 and 1:20,000, respectively. Blots were scanned with an Odyssey Infrared Imaging System (Odyssey, Li-Cor Biosciences, Nebraska, USA). Densitometric analyses of Western blot signals were performed at non-saturating exposures and analyzed using ImageJ software (National Institutes of Health, Bethesda, Maryland, USA; available at: http://rsb.info.nih.gov/ij/index.html, accessed on 12 November 2021). Values were normalized to β-tubulin, which served as a loading control.

### 2.11. Statistical Analysis

Data are represented as mean ± SEM. One-way analysis of variance was used to compare differences among groups, and statistical significance was assessed by the Tukey–Kramer post-hoc test. The level of significance for all statistical tests was set at *p* ≤ 0.05.

## 3. Results

### 3.1. ADNP Expression in Human and Rabbit Corneal Epithelium

**Figure 1:**
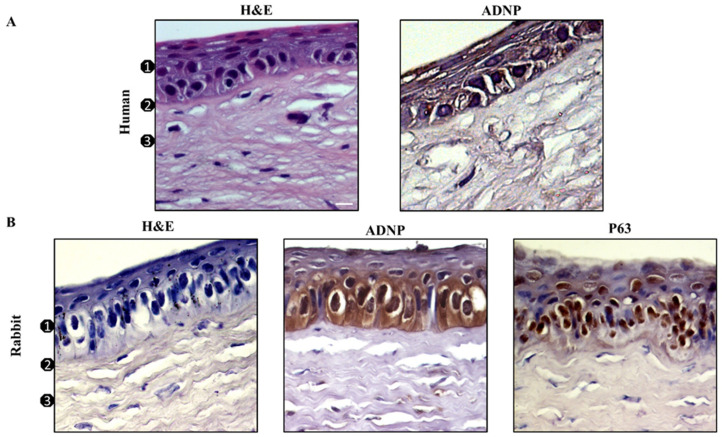
Expression of ADNP and p63 in corneal epithelium. H&E staining and immunodetection of ADNP and p63 in human (**A**) and rabbit (**B**) cornea: (1) Epithelium, (2) Bowman’s membrane, (3) stroma. Original magnification ×200. Digital micrographs are representative results of fields taken in randomly selected slides and obtained using the Zeiss Axioplan light microscope (Carl Zeiss) fitted with a digital camera (AxioCam MRc5; Carl Zeiss); scale bar: 20 μm.

The detection of ADNP expression in human and rabbit corneal epithelium was performed using IHC analysis. As shown in Figure 1, H&E staining of humans’ and rabbits’ cornea sections showed comparable architecture overall. In humans as well as in rabbits, ADNP was expressed in all examined epithelial layers, although higher staining intensity was observed in cells laying on the contact area of the basement membrane (Figure 1A,B). To identify the cellular phenotype mainly expressing the peptide, we also analyzed the distribution of the corneal epithelial stem cell marker p63. As shown in Figure 1B, ADNP was mainly expressed in the basal layer containing a large number of p63-positive cells [38].

**Figure 2:**
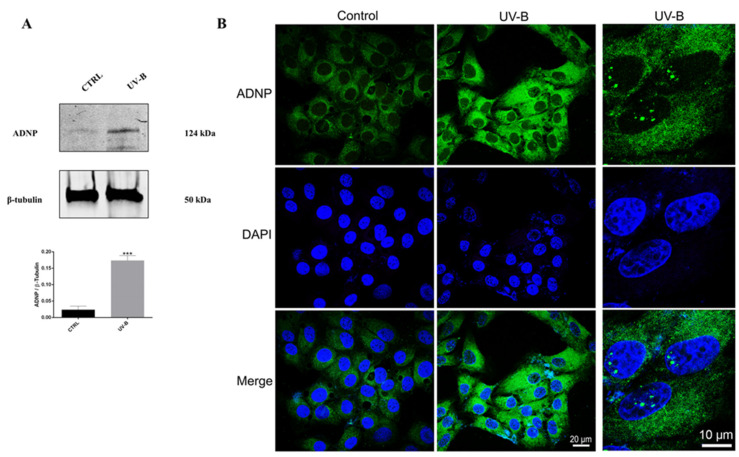
Expression of ADNP in SIRC cells exposed to UV-B radiations. (**A**) Representative immunoblots of ADNP expression in SIRC cells following exposure to UV-B insult. The bar graph shows quantitative analysis of signals obtained by immunoblots resulting from three independent experiments. Relative band densities were quantified using ImageJ software. Protein levels are expressed as arbitrary units obtained following normalization to b-tubulin, which was used as loading control. Data represent means ± SEM. *** *p* < 0.001 vs. CTRL, as determined by unpaired two-tailed Student *t*-test. (**B**) Representative photomicrographs showing ADNP expression (green) in SIRC cells exposed to UV-B radiations. Nuclei were stained with DAPI. Photomicrographs are representative results of fields taken randomly from each slide and scanned by confocal laser scanning microscopy (CLSM; Zeiss LSM700). In the right column, a thin section (0.3 µm) with some SIRC cells showing ADNP in the euchromatic compartment of the nuclei is shown. Scale bar in the left and middle columns is 20 μm, and in the right column is 10 μm.

To investigate whether ADNP expression is affected by UV-B irradiation, we analyzed the expression levels of this protein in rabbit corneal epithelial cells following exposure to UV-B insult. As shown in Figure 2A, the expression levels of ADNP were significantly induced by UV-B radiation compared to the control as measured by Western blot analysis (*** *p* < 0.001 vs. CTRL). To establish ADNP subcellular localization, immunofluorescence analysis was performed. As depicted in Figure 2B, ADNP was detected in the cytoplasm of controls. Interestingly, in SIRC cells following UV-B exposure, ADNP was revealed not only in the cytoplasm but also in the nucleus by forming small subnuclear bodies. This result suggested that UV-B radiation induced the translocation of the protein from the cytoplasm to nucleus. In accord, at the single cell level, ADNP was found both in the nucleus and in the cytoplasm, and the ADNP cytoplasm-nucleus translocation was already observed by Dr. Gozes’ group [39].

### 3.2. NAP Treatment Reduced UVB-Irradiation-Induced Apoptosis on Corneal Epithelial Cells

**Figure 3:**
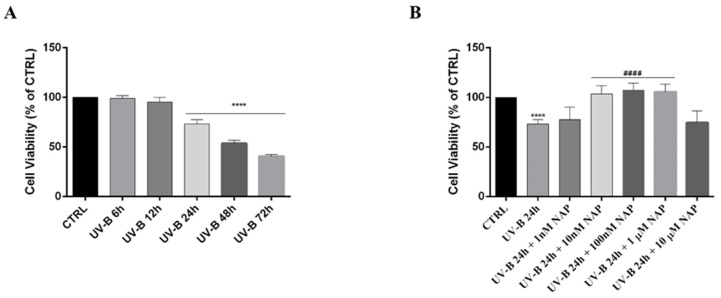
(**A**) Time–response curve following 30 s UV-B-irradiation of corneal epithelial cells. (**B**) Dose–response analysis of SIRC cells viability exposed to 30 s UV-B irradiation to various NAP concentrations for 24 h. The bar graphs represent mean ± SEM of three independent experiments and values are expressed as % of CTRL. **** *p* < 0.0001 vs. CTRL; #### *p* < 0.0001 vs. UV-B 24 h as determined by one-way ANOVA followed by Tukey’s multiple comparison test. Twelve replicate wells were used for each group.

Acute UV-B exposure provokes damage in the cornea by inducing apoptotic cell death [40,41]. To investigate the role of ADNP on corneal epithelium, we tested the effect of NAP, a small peptide mimicking the ADNP effect, in a model in vitro of corneal epithelial cells exposed to this insult. To choose the most suitable time point to test the protective action of the peptide, we performed a cell-viability time–response curve by exposing SIRC cells to 30 s UV-B-irradiation (20 mJ/cm^2^). As shown in Figure 3A, cell viability was significantly reduced in a time-dependent manner starting from 24 h following UV-B insult (**** *p* < 0.0001 vs. CTRL). Therefore, we selected this time point to perform the other experiments. In order to choose the minimum effective dose of NAP able to counteract the UV-B-irradiation-damage, we performed a dose–response analysis by treating cells with various NAP concentrations for 24 h. As shown in Figure 3B, 10 nM of NAP concentration significantly increased cell viability as compared to the UV-B group (#### *p* < 0.0001 vs. UV-B).

**Figure 4:**
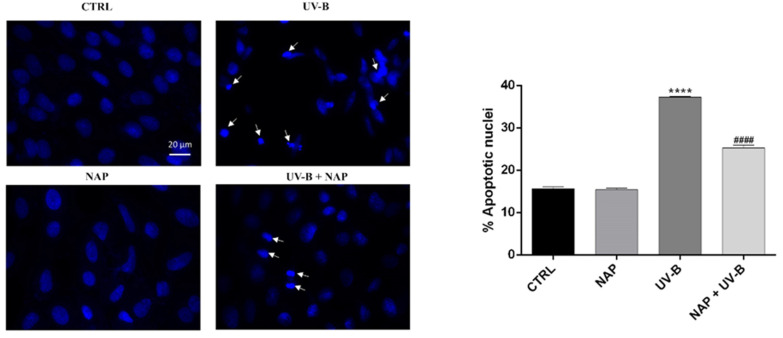
NAP decreased apoptotic cell death induced by UVB-irradiation. SIRC cells were stained with the fluorescent nuclear dye Hoechst 33342 and viewed at ×100 magnification. Nuclei showing the typical features of apoptotic degeneration including chromatin condensation and fragmentation (indicated by white arrows) have been detected by intense blue fluorescence. The bar graph represents mean ± SEM of apoptotic cells percentages calculated counting cells from seven fields per dish, in a fixed pattern. **** *p* < 0.0001 vs. CTRL; #### *p* < 0.0001 vs. UV-B as determined by one-way ANOVA followed by Tukey’s multiple comparison test. Digital micrographs are obtained with an Axiovert 40 fluorescence microscope (Carl Zeiss).

Then, we assessed the effect of NAP to prevent the apoptotic death of cells exposed to UV-B by using Hoechst 33342 staining. As shown in Figure 4, nuclei apoptotic degeneration, represented by an intense blue fluorescence due to the chromatin condensation and fragmentation, was mainly observed in the UV-B exposed group as compared to the control. The treatment of UV-B-exposed cells with NAP significantly reduced the percentage of apoptotic nuclei.

**Figure 5:**
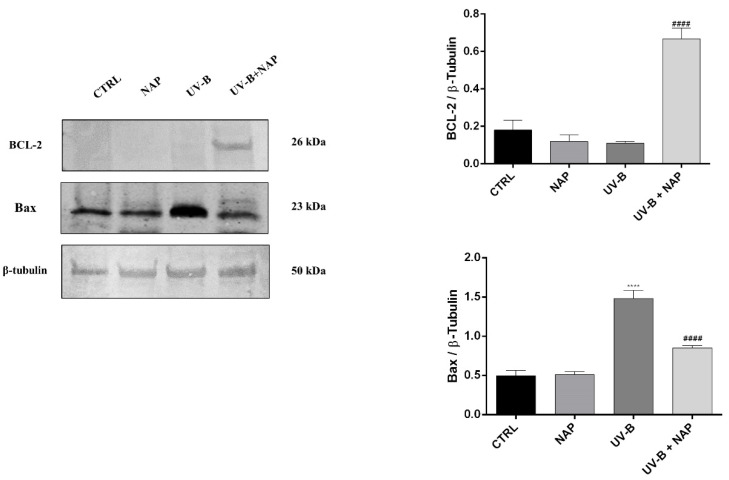
Effect of NAP on Bcl-2/Bax expression of corneal epithelial cells exposed to UV-B rays. Representative immunoblot of Bcl-2 and Bax protein expression in SIRC cells cultured in control medium (CTRL) or treated with 10 nM of NAP, or exposed to UV-B irradiation for 30 s with or without 10 nM of NAP. The bar graph shows quantitative analysis of signals obtained by immunoblots resulting from three independent experiments. Relative band densities were quantified by using ImageJ software. Protein levels are expressed as arbitrary units obtained after normalization to β-tubulin, which were used as loading control. Data represent means ± SEM. **** *p* < 0.0001 vs. CTRL; #### *p* < 0.0001 vs. UV-B as determined by one-way ANOVA followed by Tukey’s multiple comparison test.

To confirm the protective effect of NAP against UV-B-induced apoptosis, we analyzed the expression of apoptosis-associated proteins such as Bcl-2 and Bax through Western blot analysis. As shown in Figure 5, the treatment with NAP of UV-B-exposed cells significantly enhanced Bcl-2 expression. As expected, the expression levels of Bax were opposite to the Bcl-2 protein. In fact, high levels of this apoptosis-inducing protein were found in SIRC cells following UV-B exposure. The NAP treatment reduced Bax expression, restoring it to control levels (Figure 5).

### 3.3. NAP Treatment Reduced ROS Formation Induced by UV-B Irradiation of Corneal Epithelial Cells

**Figure 6:**
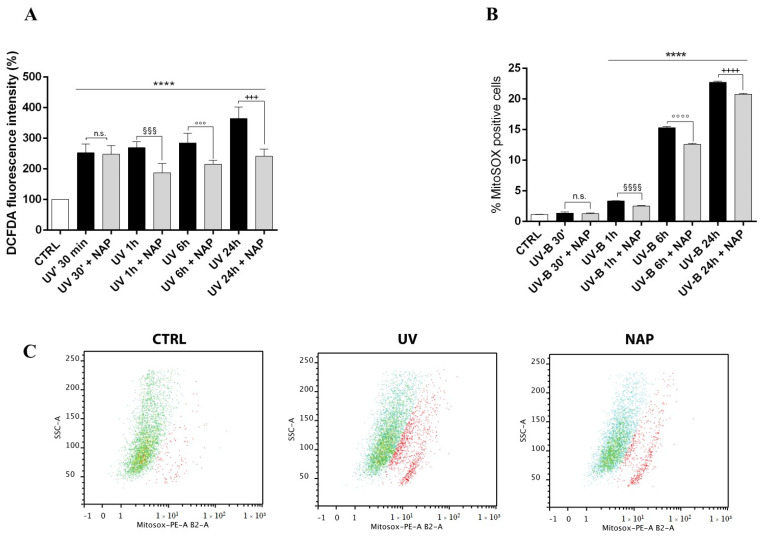
Effect of NAP on UV-B-induced ROS production on corneal epithelial cells. Intracellular ROS levels were measured in SIRC cells at different time intervals after UV-B irradiation using the cytoplasmic probe, DCFDA (**A**), or the mitochondrial probe, MitoSOX™ Red (**B**) at different time points after irradiation with or without NAP treatment. (**C**) Representative flow cytometry analysis of mitosox Red staining at 6 h. Data are expressed as mean ± SEM of three independent experiments and values are expressed as % of MitoSOX™ Red positive cells. **** *p* < 0.0001 vs. CTRL; §§§ *p* < 0.001 and §§§§ *p* < 0.0001 vs. UV 1 h; °°° *p* < 0.001 and °°°° *p* < 0.0001 vs. UV 6 h; +++ *p* < 0.001 and ++++ *p* < 0.0001 vs. UV 24 h as determined by one-way ANOVA followed by Tukey’s multiple comparison test.

It is well known that the apoptotic event induced by UV-B radiation is mediated by ROS overproduction. To evaluate whether corneal epithelial cell death was related to free radical formation following UV-B insult, we measured ROS levels at different time points in SIRC cells exposed for 30 s to UV-B irradiation (20 mJ/cm^2^) using a DCFDA assay. As shown in Figure 6A, cellular ROS levels significantly increased in a time-dependent manner starting from 30 s after UV-B irradiation. The treatment with NAP significantly reduced their formation starting from 1 h after UV-B stress. Considering that the mitochondrial superoxide anion is the major ROS formed under stress conditions and strictly involved in apoptotic cell death, we detected its time-dependent formation in cells exposed to UV-B irradiation using the MitoSOX™ Red fluorescence indicator (Figure 6B). We found that its production was increased starting from 1 h but preponderant at 6 h and 24 h after UV-B irradiation. NAP significantly decreased UV-B-induced mitochondrial ROS generation following 1 h of treatment.

**Figure 7:**
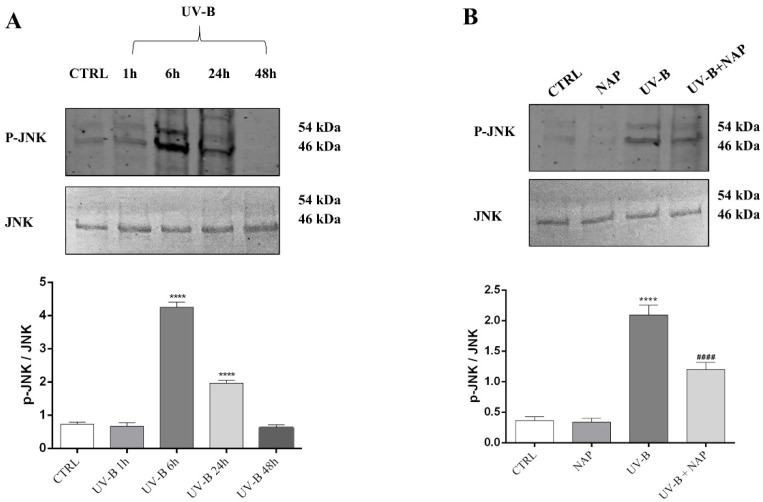
(**A**) Time-dependent effect of UV-B-induced JNK activation on corneal epithelial cells. The JNK phosphorylation was measured in SIRC cells at different time intervals after UV-B irradiation. (**B**) NAP treatment prevents UV-B-induced JNK phosphorylation in corneal epithelial cells. Representative immunoblots of JNK phosphorylation expression at 6 h in SIRC cells cultured in control medium (CTRL) or treated with 10 nM of NAP, or exposed to UV-B irradiation with or without 10 nM of NAP. The bar graphs show quantitative analysis of signals obtained by immunoblots resulting from three independent experiments. Relative band densities were quantified by using ImageJ software. Protein levels are expressed as arbitrary units obtained following normalization to total JNK, which was used as loading control. Data represent means ± SEM. **** *p* < 0.0001 vs. CTRL; #### *p* < 0.0001 vs. UV-B as determined by one-way ANOVA followed by Tukey’s multiple comparison test.

The c-Jun N-terminal kinases (JNKs), a subfamily of MAPKs, is associated with various cellular stresses by contributing to the induction of apoptosis [42]. In particular, the JNK pathway is activated by oxidative stress induced by UV-B exposure [43]. To investigate whether JNK/MAPK signaling pathway activation was involved in UV-B-ROS-induced cell death, we evaluated the time-course of p-JNK/JNK expression in cells exposed to 30 s UV-B exposure by performing Western blot analysis. As shown in Figure 7A, a significant increase in p-JNK was observed at 6 h and 24 h following UV-B insult (**** *p* < 0.0001 vs. CTRL). Therefore, we evaluated the effect of NAP on JNK activation after 6 h UV-B irradiation since, at this time point, a peak of p-JNK expression was observed. On the other hand, NAP treatment significantly reduced JNK activation in the UV-B irradiated group (Figure 7B; #### *p* < 0.0001 vs. UV-B).

## 4. Discussion

UV light is an environmental agent causing significant ocular damage and inflammation. Sunlight with a wavelength shorter than 295 nm is absorbed by the ozone layer, whereas UV-B (290–320 nm) and UV-A (320–400 nm) radiations can reach the cornea and lens, respectively. The UV-B-induced corneal injury is due to oxidative stress induced by ROS formation in the corneal epithelial cells [44,45]. ROS, including the superoxide anion, hydrogen peroxide, and hydroxyl radicals, cause DNA mutations as well as apoptotic cell death. In particular, a single acute dose of UV-B rays could damage the corneal epithelium, whereas higher doses led to a remarkable decrease in its thickness with a significant reduction of Na^+^-K^+^-dependent adenosine triphosphatase, representing the major transporter involved in the corneal hydration [46,47,48,49,50]. Several papers have demonstrated that NAP, a small fragment derived from the glial cell mediator ADNP, has protective effects in different ocular diseases [23,51,52]. Moreover, in vitro and in vivo studies showed that NAP protected cells against toxicity associated with oxidative stress [22], conferred protection from apoptosis [23], and promoted antioxidant defense by reducing ROS generation [53]. Since ROS formation and apoptotic cell death represent the two central features of UV-B damage [54,55], the main goal of the current study was to evaluate whether ADNP, a peptide constitutively expressed in the corneal epithelium, played a protective role against UV-B insult.

In this work, we showed, for the first time, the presence of ADNP in human and rabbit corneal epitheliums (Figure 1). ADNP-like immunoreactivity was detected in all layers of corneal epithelium of both species, although higher staining intensity was observed in the inner basal layer (Figure 1A,B). The latter is formed by a stem cell monolayer involved in the regeneration of the corneal epithelium [56] as confirmed by p63 immuno-positive staining. These data suggested that ADNP might have an important role in corneal regeneration. To investigate its protective role on the corneal epithelium, we performed an in vitro study on SIRC cells exposed to UV-B irradiation. Here, we found high expression levels of the ADNP protein in cells exposed to UV-B rays for 30 s (Figure 2A). Moreover, immunofluorescence analysis revealed that ADNP was detected not only in the cytoplasm but also in the nucleus by forming small subnuclear bodies (Figure 2B). Various literature data have demonstrated nuclear localization of ADNP [57,58]. This protein contains the thiotransferase/glutaredoxin active site [22], which regulates its own and other DNA-binding proteins’ expression in response to oxidative stress. Therefore, ADNP translocation from the cytoplasm to the nucleus and vice versa could be involved in the mechanism regulating cell fate in response to UV-B damage.

To investigate whether higher expression of the ADNP protein following the UV-B irradiation is related to its protective role against this insult, we treated irradiated cells with the ADNP-derived small active peptide, NAP, whose structure allows membrane penetration. Many studies demonstrated that this small active element is able to protect cells against the induction of cell death by a variety of stresses [21,22,23,24,25,26,27,59]. Here, we demonstrated that NAP treatment prevents UV-B rays-induced damage (Figure 3B) by reducing the percentage of apoptotic cells (Figure 4). To confirm these data, we also evaluated the expression of two proteins: The proapoptotic protein Bax and the antiapoptotic protein Bcl-2, both implicated in the maintenance of tissue homeostasis. Under physiological conditions, their expression is in equilibrium, whereas exposure to different cytotoxic stimuli, such as UV-B radiation, alter this balance leading to apoptotic cell death via an increase in Bax levels. In accord with this evidence, our data indicated that UV-B-irradiation results in a significant upregulation of Bax expression (Figure 5). NAP treatment prevented apoptotic death of irradiated cells by increasing Bcl-2 and decreasing BAX levels (Figure 5). These findings were consistent with previous reports describing that NAP displayed anti-apoptotic activity through the downregulation of BAX and upregulation of Bcl-2 expression following exposure to different type of insults such as early diabetic injury or hyperglycemic/hypoxic events [28,60]. The UV-B radiations damage is mediated by ROS overproduction, which plays a key role in apoptotic cell death induction [54,55]. In accord, our results showed that UV-B radiation markedly increased cellular ROS generation as well as mitochondrial superoxide anion formation in a time-dependent manner. NAP treatment exerted a regulatory effect on UV-B-induced oxidative stress by counteracting ROS overproduction and, in particular, O_2_^•−^ formation in the mitochondria (Figure 6A and B).

In corneal epithelial cells exposed to UV-B, the increase in ROS induced apoptotic cell death through the activation of the JNK MAPK pathway. It is well known that ROS formed during UV-B exposure acts as a second messenger by activating the JNK MAPK signal transduction pathway [59,61]. The activation of this signaling cascade is directly involved in apoptotic cell death. In fact, it has been previously demonstrated that p-JNK binds to a pro-apoptotic Bcl-2 member, known as Bim, activating Bax-dependent apoptotic machinery during cells’ exposure to UV-B [62,63]. Here, we confirmed that UV-B rays trigger JNK phosphorylation, whereas its activation is abrogated by NAP treatment (Figure 7). These results suggested that NAP may counteract UV-B-induced apoptotic cell death by modulating ROS production and antagonizing the JNK MAPK pathway.

**Figure 8:**
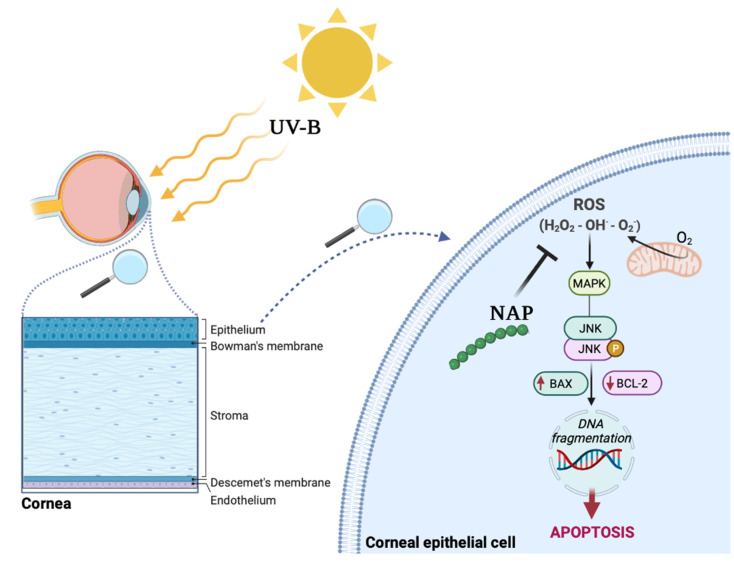
Protective effects of NAP on corneal epithelium exposed to UV-B radiation. Schematic representation of NAP effect on ROS formation, indirectly inducing downregulation of JNK/Bax signaling pathways.

The exact step in the UV-B-induced apoptotic cell death regulated by NAP is still unclear. However, we hypothesized that its effect is related to the upstream reduction in ROS formation. It has been previously reported that NAP has antioxidant properties by interfering with various cellular insults [28,54]. Therefore, the preferential way travelled by NAP could lead to the reduction of UV-B-induced ROS formation by indirectly inhibiting the JNK/Bax signaling pathway. The mechanism underlying the proposed connections is represented in Figure 8.

One of the weaknesses of this study is that experiments are performed on the SIRC cell line growing as a single monolayer. This experimental approach does not allow one to simulate all the in vivo properties of the corneal stratified squamous epithelium. Despite this limitation, the data reported in the present paper suggested that NAP might serve as a potential therapeutic agent for corneal epithelial damage attributable to its antioxidant and anti-apoptotic activity. Therefore, this evidence should prompt the scientific community to further investigate NAP effects in the cornea in vivo exhibiting a multi-layered complex architecture.

## Data Availability

The data presented in this study are available in article.
